# ID 29 - Saúde e Educação no Acesso ao Conhecimento sobre ATS

**DOI:** 10.5327/2237-9622.2025.v34s1.197

**Published:** 2025-11-25

**Authors:** Mariana Dias Lula, Jéssica Evelyn de Andrade, Juliana Álvares Teodoro, Augusto Afonso Guerra, Cristina Mariano Ruas

## Abstract

**Introdução:**

O Saúde e Educação (SeE) é um projeto de extensão da Faculdade de Farmácia da Universidade Federal de Minas Gerais (UFMG), que objetiva divulgar informações e pesquisas relacionadas à saúde de forma simplificada e compreensível para o público geral. A partir da percepção de lacunas de conhecimento existentes na sociedade, o SeE idealizou uma série temática, com o intuito de divulgar informações claras e acessíveis sobre a Avaliação de Tecnologias em Saúde (ATS) e sobre o trabalho executado pelos Núcleos de Avaliação de Tecnologias em Saúde (Nats). Para atingir esse objetivo, foi realizada uma parceria com o Centro Colaborador do SUS para Avaliação de Tecnologias e Excelência em Saúde (Ccates/UFMG). Membros do Ccates foram convidados pelo SeE a produzir, de forma voluntária, textos informativos sobre ATS para serem publicados e divulgados pelo SeE. Os textos passam por revisão das editoras do site. Um dos pontos avaliados durante a revisão é que o material produzido tenha estrutura e linguagem de fácil compreensão ao público leigo, sem perder a qualidade técnica. Uma vez revisados e ajustados, os textos passam por formatação para que fiquem com leiaute que motive a leitura e facilite a compreensão pelo leitor. Então, os materiais são publicados no site e divulgados nas redes sociais do projeto. As artes das publicações nas redes sociais são elaboradas com elementos textuais e não textuais, como fotos do autor e figuras relacionadas. A divulgação é feita pela equipe do SeE e os autores são incentivados a contribuir com a divulgação. São selecionados dias estratégicos para a publicação, como datas comemorativas, e incluídas hashtags de interesse. Parcerias com associações de pacientes e outros perfis de divulgação científica, por exemplo, são realizadas a favor da divulgação. Como resultado, no ano de 2024, três textos, de cinco colaboradores, foram produzidos, publicados e divulgados. Quatro estão em processo de elaboração e/ou revisão. Até o momento, os textos obtiveram, ao todo, 132 visualizações no site; e as divulgações, mais de mil visualizações nas redes sociais. Desafios encontrados incluem a tradução do conhecimento e o alcance do público-alvo. Essa iniciativa tem relevância para sistemas de saúde, visto que a democratização da ATS e a divulgação científica, especialmente através de meios digitais, são importantes para garantir que as informações sejam acessíveis a todos os atores envolvidos, incluindo gestores, profissionais de saúde, pacientes e o público em geral. Isso promove uma tomada de decisão mais inclusiva e participativa, permitindo que diferentes perspectivas sejam consideradas, aumenta a transparência e a confiança no processo, contribui para uma maior simetria de informações e equidade no acesso a inovações em saúde, além de fortalecer o controle social e melhorar a implementação de tecnologias que atendam às reais necessidades da população. A iniciativa será apresentada em forma de comunicação oral, com uso de eslaides.

**Método:**

A apresentação compreenderá as seguintes etapas: i) iniciativa: descrição da iniciativa e metodologia para alcance dos resultados; ii) resultados: descrição dos resultados alcançados com a iniciativa; e iii) discussão: discussão da iniciativa, a importância dos resultados alcançados e as perspectivas futuras. Os equipamentos necessários para a apresentação serão: computador com acesso à internet e leitor de .pdf. Projetor conectado ao computador.

